# Enhanced glycolysis contributes to the pathogenesis of experimental autoimmune neuritis

**DOI:** 10.1186/s12974-018-1095-7

**Published:** 2018-02-21

**Authors:** Ru-Tao Liu, Min Zhang, Chun-Lin Yang, Peng Zhang, Na Zhang, Tong Du, Meng-Ru Ge, Long-Tao Yue, Xiao-Li Li, Heng Li, Rui-Sheng Duan

**Affiliations:** 1 Department of Neurology, Shandong Provincial Qianfoshan Hospital, Shandong University, Jinan, 250014 People’s Republic of China; 2 Central Laboratory, Shandong Provincial Qianfoshan Hospital, Shandong University, Jinan, 250014 People’s Republic of China

**Keywords:** Experimental autoimmune neuritis, Glycolysis, 2-Deoxy-d-glucose, Inflammation

## Abstract

**Background:**

With the recognition of the key roles of cellular metabolism in immunity, targeting metabolic pathway becomes a new strategy for autoimmune disease treatment. Guillain-Barré syndrome (GBS) is an acute immune-mediated inflammatory demyelinating disease of the peripheral nervous system, characterized by inflammatory cell infiltration. These inflammatory cells, including activated macrophages, Th1 cells, and Th17 cells, generally undergo metabolic reprogramming and rely mainly on glycolysis to exert functions. This study aimed to explore whether enhanced glycolysis contributed to the pathogenesis of experimental autoimmune neuritis (EAN), a classic model of GBS.

**Methods:**

Preventive and therapeutic treatments with glycolysis inhibitor, 2-deoxy-d-glucose (2-DG), were applied to EAN rats. The effects of treatments were determined by clinical scoring, weighting, and tissue examination. Flow cytometry and ELISA were used to evaluate T cell differentiation, autoantibody level, and macrophage functions in vivo and in vitro.

**Results:**

Glycolysis inhibition with 2-DG not only inhibited the initiation, but also prevented the progression of EAN, evidenced by the improved clinical scores, weight loss, inflammatory cell infiltration, and demyelination of sciatic nerves. 2-DG inhibited the differentiation of Th1, Th17, and Tfh cells but enhanced Treg cell development, accompanied with reduced autoantibody secretion. Further experiments in vitro proved glycolysis inhibition decreased the nitric oxide production and phagocytosis of macrophages and suppressed the maturation of dendritic cells (DC).

**Conclusion:**

The effects of glycolysis inhibition on both innate and adaptive immune responses and the alleviation of animal clinical symptoms indicated that enhanced glycolysis contributed to the pathogenesis of EAN. Glycolysis inhibition may be a new therapy for GBS.

## Background

Guillain-Barré syndrome (GBS) is an acute immune-mediated peripheral neuropathy, characterized by rapidly progressive motor and sensory dysfunction in the limbs, dysfunction of autonomic nervous system and respiratory failure [1]. It is considered that both humoral and cellular immune responses are involved in the pathogenesis of GBS, but the exact mechanisms are still not clear. Despite most patients have good outcomes after conventional treatments like plasma exchange and intravenous immunoglobulin therapy, 3–10% of patients still die and 20% are still unable to walk after 6 months [1]. Therefore, more acceptable and efficacious therapy is needed. Experimental autoimmune neuritis (EAN) is the mostly used animal model of GBS for exploring the underlying pathogenesis and developing new therapies. This model could be induced in susceptible animal species with myelin proteins or synthetic peptides of myelin proteins emulsified in complete Freund’s adjuvant [2].

Over the past decades, the field of immunology has been focused on the genetic and signaling programs during immune responses. Only recently, were the concomitant reprogramming of metabolic pathways established as a key component of immune cell regulation and function [3]. In the innate immune system, once activated by the pro-inflammatory stimuli, macrophages and dendritic cells (DC) undergo a metabolic switch away from oxidative phosphorylation (OXPHOS) towards glycolysis [4, 5] even in the presence of abundant oxygen, similar to the Warburg effect observed in cancer cells [6]. The underlying mechanisms include the upregulation of inducible nitric oxide synthase (iNOS) and the subsequent NO-mediated mitochondrial respiration, the activation of mammalian target of rapamycin (mTOR)-hypoxia-inducible factor-1α (HIF-1α) pathway and the subsequent upregulation of glycolysis associated genes, as well as the inhibition of AMP-activated protein kinase (AMPK) and subsequent downregulation of β-oxidation of fatty acids and mitochondrial biogenesis [7]. In the adaptive immune system, different cells also display different metabolisms. Th1, Th2, and Th17 cells tend to be more glycolytic, while regulatory T (Treg) cells rely more on lipid metabolism [8].

It is believed that high glycolysis not only meets the increased energy demand but also provides adequate biosynthetic precursors for proteins, lipids, and nucleic acids in the process of cell activation, proliferation, and effector function. Moreover, some of the metabolites even function as signaling molecules, like citrate in the epigenetic activation [9], succinate in the stability of HIF-1α, and subsequent IL-1β secretion [10]. The different metabolic demands of different cells provide a promising opportunity for selective regulation of immune subsets. Targeting metabolism pathway becomes a new strategy in autoimmune disease treatment [3].

Although the enhanced glycolysis has been reported in systemic lupus erythematosus (SLE) [11] and autoimmune arthritis [12, 13], up to now, no research about the metabolic reprogramming in GBS or EAN can be found. Considering the hypermetabolism and remarkable weight loss in GBS patients [14] and the fact that fasting glucose levels correlate with the disease severity of GBS [15], it is plausible to assume that enhanced glycolysis exists and contributes to the pathogenesis of GBS. Thus, we explored the roles of glycolysis in the classic model of EAN with a well-known glycolysis inhibitor, 2-deoxy-d-glucose (2-DG) [10, 16–18]. As a glucose analogue, 2-DG is phosphorylated by hexokinase (HK) to 2-DG-phosphate which cannot be further metabolized. The accumulated 2-DG-phosphate leads to the inhibition of glycolysis [18].

In this study, we applied 2-DG in preventive and therapeutic patterns and evaluated clinical scores, pathological changes and cellular and humoral immune responses in vivo. Effects of glycolysis inhibition on DC and macrophages were also explored in vitro*.*

## Methods

### Materials

RAW264.7 cells were purchased from American Type Culture Collection (ATCC) and routinely cultured in high glucose DMEM (glucose 4.5 g/l) supplemented with 10% fetal bovine serum (FBS) and 1% penicillin–streptomycin. Bovine peripheral myelin (BPM) was prepared according to our previous report [2]. 2-DG was purchased from Aladdin (Aladdin, Shanghai, China). DMEM was purchased from Corning (China), RPMI 1640 medium from Gibco (China), FBS from BI (Israel), and penicillin–streptomycin from Hycolone (USA).

### Measurement of glucose uptake by 2-NBDG

To obtain activated macrophages, peritoneal macrophages were first stimulated by 50 ng/ml lipopolysaccharides (LPS) (Sigma-Aldrich, USA) for 18 h. Then, 2 × 10^5^ resting or activated peritoneal macrophages were incubated in glucose-free RPMI medium containing 50 μM fluorescent d-glucose analogue 2-(*N*-(7-nitrobenz-2-oxa-1,3-diazol-4-yl)amino)-2-deoxyglucose (Cayman Chemical, USA) for 1 h at 37 °C [19]. Mean fluorescence intensity (MFI) of 2-NBDG in the peritoneal macrophages was analyzed by flow cytometry.

After activation of peritoneal macrophages by LPS with or without 2-DG (2 mM, 4 mM) for 18 h, the culture medium was harvested for the examination of glucose by glucometer and pH values by pH indicator.

### Experimental animals

All of the experimental protocols with rats have been approved by the institutional ethics committee of Shandong University. Lewis rats (130–230 g, 6–9 weeks old) were purchased from Vital River Laboratories (Beijing, China) and kept at the local pathogen-free animal house with free access to food and water.

### Induction of EAN and evaluation of clinical signs

To induce EAN, 200 μl inoculum containing 1 mg BPM was injected into the base of tail subcutaneously. The BPM was dissolved in 100 μl saline and then emulsified with 100 μl incomplete Freud adjuvant (Sigma-Aldrich, USA) containing 0.3 mg *Mycobacterium tuberculosis* (strain H37RA; Difco, Detoit, MI, USA). The rats were observed, weighted, and assessed by two researchers daily in a blinded fashion after immunization. Clinical scores were graded as follows: 0 = normal, 1 = reduced tonus of the tail, 2 = partial tail paralysis, 3 = complete tail paralysis or absent righting reflex, 4 = gait ataxia, 5 = mild paresis of the hind limbs, 6 = moderate paraparesis, 7 = severe paraparesis of the hind limbs, 8 = tetraparesis, 9 = moribund, and 10 = death.

### 2-DG treatment

In the preventive experiment, 2-DG was dissolved in ddWater (sterilized) to a final concentration of 60 mg/ml and given to rats (90 mg/rat) via intraperitoneal injection (i.p.). The rats in control group received the same volume of ddWater in the same way. 2-DG solution was administered daily from the day of immunization to day 13 post-immunization (p.i.) when the symptoms peaked. For therapeutic treatment, with the development of EAN, rats displayed the different degrees of neurological deficit and the weight loss between and in both groups; to minimize the influence of weight loss, 2-DG was given daily at the dose of 550 mg/kg from day 10 p.i. when the first clinical sign appeared to day 21 p.i. when all rats showed signs of recovery. Serum, inguinal lymph nodes, spleens, and sciatic nerves were harvested for further study.

### Histopathological assessment

Sciatic nerves from both groups were harvested and fixed with 4% paraformaldehyde. After dehydration using graded ethanol and vitrification with dimethylbenzene, the nerves were embedded in paraffin and sliced longitudinally into 4-μm-thick sections (Leica RM2235). Hematoxylin-eosin (H&E) and luxol fast blue (LFB) stainings were applied separately to evaluate the extent of inflammatory cell infiltration and demyelination. To quantify the inflammatory infiltration, infiltrating mononuclear cells, mostly with round or oval nuclei, were counted at × 200 magnification for three fields of each slide artificially. Results are analyzed with the average cell number per field (× 200 magnification). To evaluate the severity of demyelination, histological scores were acquired according to a semiquantitative grading system as previously reported [20]: 0 = normal, 1 = demyelinated fibers less than 25%, 2 = demyelinated fibers 25–50%, 3 = demyelinated fibers 50–75%, and 4 = demyelinated fibers more than 75%.

Immunohistochemistry was performed to investigate the macrophage infiltration. Briefly, 4-μm-thick sections described above were deparaffinized. After antigen retrieval, endogenous peroxidase elimination, and blocking with 10% FBS in PBS, mouse anti-CD68 (abcam, USA) was applied for macrophage detection at 4 °C overnight. Thereafter, horseradish peroxidase-labeled anti-mouse IgG secondary antibody (ZSGB-BIO, China) and DAB peroxidase substrate kit (ZSGB-BIO, China) were used for the subsequent staining. Positive cells were counted at × 200 magnification for three fields of each slide artificially. Results were analyzed with the average positive cell number per field (× 200 magnification).

### Flow cytometry

For mononuclear cell (MNC) preparation, spleens and bilateral inguinal lymph nodes were harvested and grinded through the cell strainer (70 μm, biologix, USA) in RPMI 1640 Medium. Osmotic lysis method was used to deplete the erythrocytes.

For T helper cell (Th) detection, MNCs from the bilateral inguinal lymph nodes were first incubated in the medium containing cell stimulation cocktail plus protein transport inhibitors (eBioscience, USA) for 5 h at 37 °C. Then, MNCs were collected and washed. FITC-conjugated anti-rat CD4 antibody (eBioscience, USA) was used to detect the cell surface antigen, and then, cells were fixed with 2% paraformaldehyde, permeabilized with permeabilization wash buffer (Biolegend, USA) according to the reagent instructions. Thereafter, APC-conjugated anti-IL-17 (eBioscience, USA) and eFluor 660-conjugated anti-IFN-γ (eBioscience, USA) were added to detect the respective antigen for 30 min at 4 °C.

Follicular helper T cells (Tfh cells) were detected by PE-conjugated anti-CD4 (Biolegend, USA), PE-Cy7-conjugated anti-ICOS (Biolegend, USA), rabbit anti-CXCR5 (Abcam, USA), and Alexa Fluor 488-conjugated anti-rabbit IgG (Abcam, USA) in the spleen MNCs. For Treg cell detection, surface staining with FITC-conjugated anti-CD4 (eBioscience, USA) and/or PE-conjugated anti-CD25 (eBioscience, USA) was first performed in the lymph node MNCs and after fixation and permeabilization, Alexa Fluor 647-conjugated anti-foxp3 (eBioscience, USA) or PE-conjugated anti-foxp3 (eBioscience, USA) was added according to the instruction manual.

DC was characterized by Alexa Fluor 647-conjugated anti-OX62 (Biolegend, USA) or PE-conjugated anti-OX62 (eBioscience, USA) in the spleen MNCs. Then, APC-anti-MHC II (eBioscience, USA) or FITC-anti-MHC II (Biolegend, USA), PE-anti-CD80 (eBioscience, USA), FITC-anti-CD86 (eBioscience, USA) or PE-anti-CD86 (Biolegend, USA) were used to detect the maturation of DC.

### Anti-BPM antibody by ELISA

Flat-bottomed polystyrene 96-well plates (Corning, USA) were coated with 100 μl BPM (10 μg/ml in PBS) overnight at 4 °C. Then, the plates were blocked with 10% FBS. Diluted serum (1: 100) was added and incubated for 2 h at 37 °C, followed by biotin-labeled anti-rat IgG (Biolegend, USA) for 1 h at 37 °C and streptavidin–horseradish peroxidase (Bios, China) for 30 min at 37 °C. The color was developed with tetramethylbenzidine (TMB) substrate (Tiangen Biotechnology, China). Finally, OD values of corresponding wells were determined by a microplate ELISA reader at 450 nm. Results were expressed as mean optical density (OD values) ± standard deviation (SD).

### DC and peritoneal macrophages preparation

To acquire DC, bone marrow cells were induced as we previously reported [21]. Firstly, femurs and tibias were aseptically removed from unimmunized Lewis rats and bone marrow cells were flushed out. After elimination of erythrocytes by osmotic lysis, the remained cells were cultured in RPMI 1640 medium supplemented with 10% FBS and 1% penicillin–streptomycin containing 10 ng/ml recombinant rat GM-CSF (Peprotech, USA) and 10 ng/ml IL-4 (Peprotech, USA) for 3 days. Then, the non-adherent cells were gently removed and the adherent cells were further cultured. After culture for another 4 days, the floating cells, namely DC, were collected. DC was further activated by 1 μg/ml LPS for 20 h with or without 2-DG (1 mM). Expression of MHC II, CD80, and CD86 were detected by flow cytometry (FC) as described above.

To obtain peritoneal macrophages, rats were first intraperitoneally injected with 3 ml of 6% starch solution. After 3 days, the rats were killed by an overdose of isoflurane (RWD Life Science, China), followed with intraperitoneal injection of 10 ml PBS. Then, after light rolling for 1 min, fluid in the abdominal cavity was withdrawn and centrifuged and the cell pellet was resuspended in complete media containing RPMI 1640 medium supplemented with 10% FBS and 1% penicillin–streptomycin. These peritoneal exudate cells were incubated for 2 h; then, non-adherent cells were removed and the adherent cells, namely macrophages, were prepared for further studies.

### Phagocytosis assay in vitro

To quantify the phagocytosis ability of macrophages, the uptake of FITC-dextran (molecular weight 40,000) was detected by flow cytometry according to the previous report [22]. Briefly, the isolated peritoneal macrophages were treated with or without 2-DG (2 mM, 4 mM) for 16 h in 24 hole cell culture plate. Then, the cells were washed with PBS and incubated with 300 μl RPMI 1640 medium containing 0.4 mg FITC-Dextran (Sigma, USA) for 1 h at 37 or 4 °C. After digestion with trypsin, the cells were harvested, washed, and analyzed by FC. For RAW264.7 cells, LPS (50 ng/ml) with or without 2-DG (4 mM) were applied for 20 h. Then, cells were harvested and treated by 150 μl DMEM containing 0.2 mg FITC-Dextran for 1 h. Cells were washed three times with PBS, and the MFI was determined by FC.

### NO production assay in vitro

For NO production assay, the isolated peritoneal macrophages were stimulated by LPS (50 ng/ml) with or without 2-DG (1 mM, 4 mM) in PRMI 1640 complete medium or glucose-higher medium (glucose concentration increased 4 g/l) for 20 h. RAW264.7 cells were activated by 50 ng/ml LPS with or without 2 mM 2-DG in DMEM complete medium or glucose-higher medium (glucose concentration increased 8 g/l) for 16 h. All the above culture medium was harvested and examined with nitric oxide (NO) assay kit (Beyotime, China) according to the instruction manual. Results were expressed as OD values ± SD.

### TNF-α secretion in vitro by ELISA

To analyze the effects of glycolysis inhibition on TNF-α secretion, the peritoneal macrophages were pretreated with 1 mM 2-DG for 3 h, followed by 50 ng/ml LPS addition for another 20 h, according to the previous study [23]. The culture medium was harvested and stored at − 20 °C. The levels of TNF-α were examined with anti-rat TNF-α ELISA kit (eBioscience, USA) according to the instruction manual. Results were expressed as OD values ± SD.

### Statistical analysis

Statistical analysis was performed with GraphPad Prim 6.0. Differences between two groups were tested by two-tailed Student *t* test and among three groups by one-factor analysis of variance (ANOVA). Data were expressed as mean ± SD, and *p* < 0.05 was considered significant.

## Results

### 2-DG inhibited the increased glycolysis in LPS-stimulated peritoneal macrophages

**Fig. 1:**
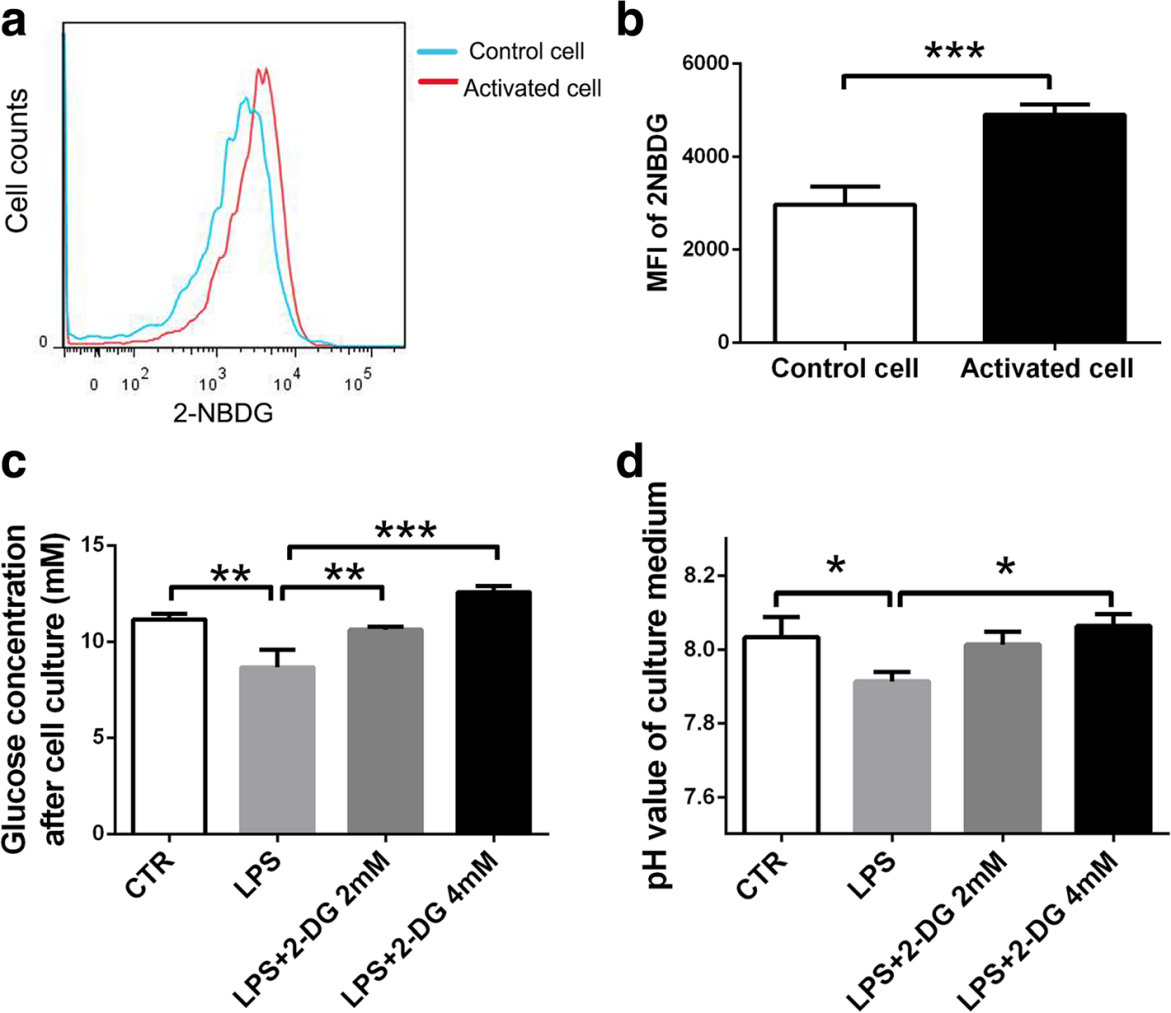
2-DG inhibited the increased glycolysis in LPS-stimulated peritoneal macrophages in vitro. **a** Uptake of the fluorescent glucose analogue 2-NBDG in the LPS-stimulated peritoneal macrophages, measured by flow cytometry. **b** Graphic representation of the mean fluorescence intensity shown in (**a**). ***p < 0.001, *n* = 3, two-tailed Student’s *t* test for unpaired data. Surplus glucose (**c**) and pH values (**d**) of the culture supernatants after stimulating peritoneal macrophages by LPS (50 ng/ml) with or without 2-DG for 18 h. *p < 0.05, **p < 0.01, ***p < 0.001, *n* = 4, one-factor analysis of variance (ANOVA). The results are expressed as mean ± SD

Macrophages represent the major effector cell in the inflamed peripheral nerves of EAN and are responsible for most of the neuropathological destruction. To examine whether enhanced glycolysis exists in the activated macrophages, we accessed the metabolism of LPS-stimulated peritoneal macrophages in vitro. Using fluorescence-labeled glucose analogue, 2-NBDG, we found that after stimulation, glucose uptake was significantly increased in the peritoneal macrophages (*p* < 0.001) (Fig. 1a, b). Meanwhile, the glucose concentration in the culture medium, which revealed the glucose consumption, dropped sharply (*p* < 0.01) (Fig. 1c), and the pH values, which reflected the level of lactic acid, declined significantly (*p* < 0.05) (Fig. 1d). The changes of glucose uptake, glucose consumption, and pH values all indicated an enhanced glycolysis in the activated macrophages. After 2-DG (4mM) treatment, the enhanced glucose consumption and acidity of culture medium were reduced significantly (*p* < 0.001, *p* < 0.05, respectively) (Fig. 1c and d).

### 2-DG inhibits the initiation of EAN

**Fig. 2:**
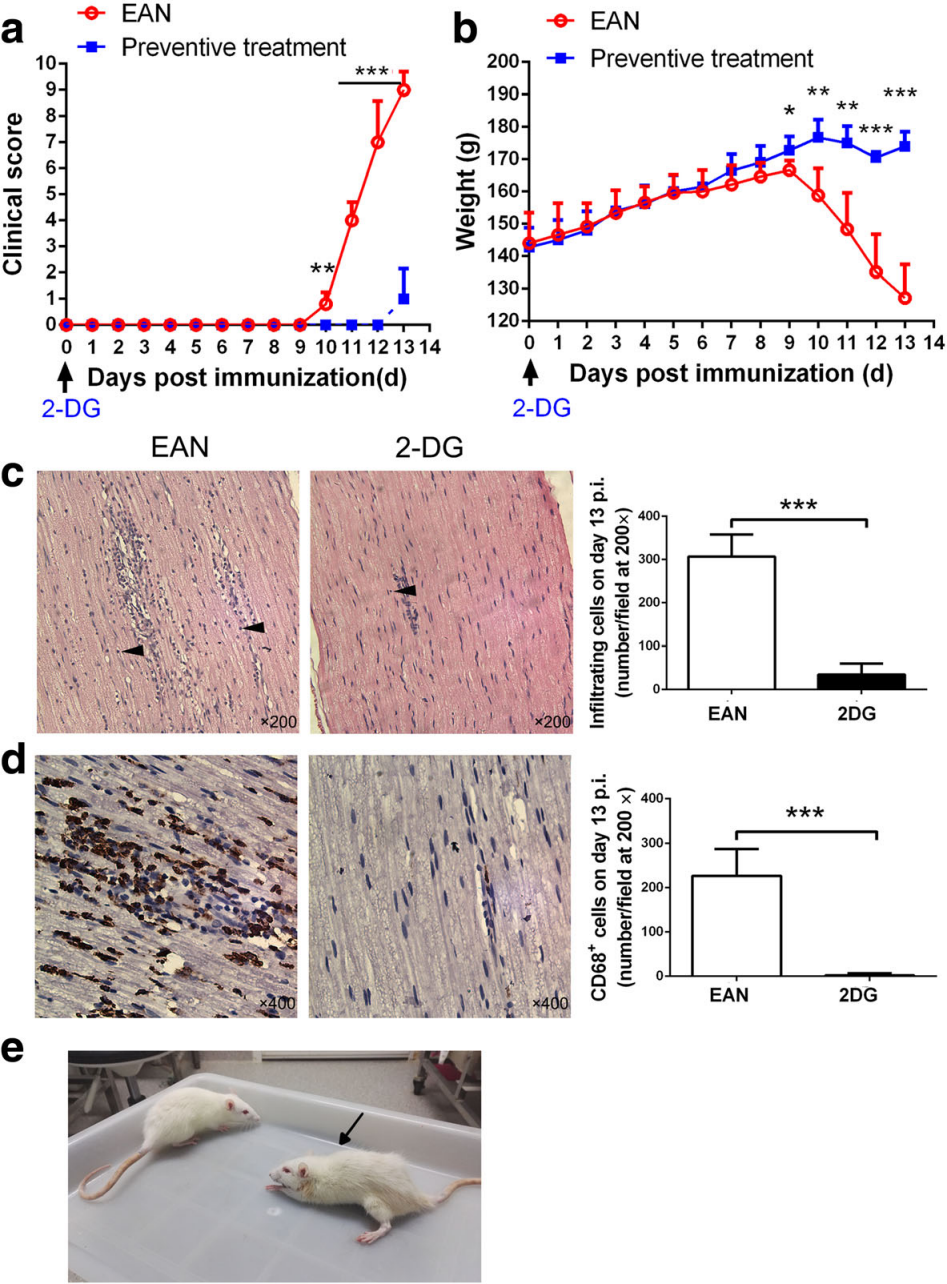
2-DG inhibited the initiation of EAN. 2-DG (90 mg/rat) was intraperitoneally injected to BPM-induced EAN rats daily starting from the day of immunization to day 13 p.i. when the symptoms peaked. *n* = 4 in 2-DG group; *n* = 5 in the control group (one died of respiratory failure before the end point). Clinical scores (**a**) and body weight (**b**) were evaluated and recorded every day. *p < 0.05, **p < 0.01, ***p < 0.001. Sciatic nerves were harvested on day 13 p.i. for H&E staining (**c**) and CD68 immunostaining (**d**) (*n* = 4 in each group). The numbers of the total infiltrated mononuclear cells and macrophage (brown) at × 200 magnification were analyzed and displayed (***p < 0.001) (arrows denote the inflammatory mononuclear cells). The representative rats from both groups on day 12 p.i. were shown in (**e**). Compared with the 2-DG-treated rat, the rat from control group (arrowed) showed more serious weight loss and paralysis. The results are expressed as mean ± SD, two-tailed Student’s *t* test for unpaired data

To explore the role of glycolysis in the initiation of EAN, the rats were treated with 2-DG (90 mg/rat) daily from the day of immunization. The rats in the control group showed weakness on day 10 post-immunization (p.i.), and the symptoms progressed rapidly and peaked on day 13 p.i. (mean clinical score 9 ± 0.71), while the rats in 2-DG group showed the mild neurological signs on day 13 p.i. (mean clinical score 1 ± 1.15) (Fig. 2a). With the progression of EAN, the control rats suffered progressive weight loss from day 9 p.i. to day 13 p.i. (mean weight loss 39.4 ± 8.02 g). In contrast, no significant weight loss was observed in 2-DG-treated rats during this period (mean weight loss − 2 ± 3 g) (Fig. 2b). A 2-DG-treated rat showed milder symptom compared with a control rat (arrowed) on day 12 p.i. (Fig. 2e). Therefore, preventive treatment of 2-DG significantly inhibited the initiation of EAN.

Then, we further examined the sciatic nerves on day 13 p.i. In the sciatic nerves of the EAN rats, the mainly infiltrated inflammatory cells were macrophages and CD4^+^ T cells, mostly with round or oval nucleus. We found 2-DG treatment significantly reduced the inflammatory cell infiltration determined by H&E staining (*p* < 0.001) (Fig. 2c), especially the infiltration of CD68^+^ macrophages determined by immunohistochemistry (*p* < 0.001) (Fig. 2d).

### Preventive treatment of 2-DG modulates both cellular and humoral immune response in vivo

**Fig. 3:**
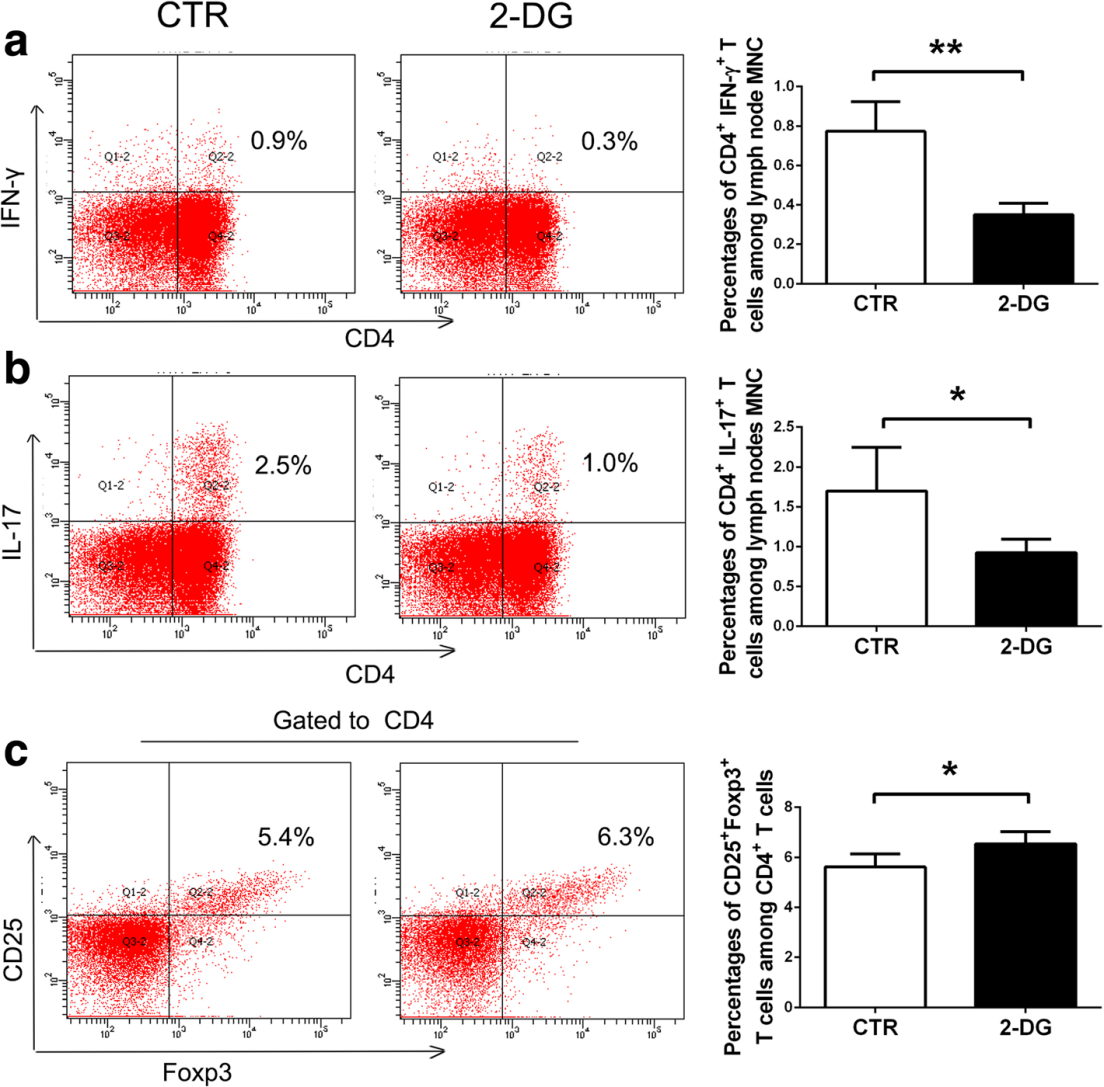
Preventive treatment of 2-DG decreased Th1 and Th17 cells but increased Treg cells in vivo. MNCs of the lymph nodes were harvested from both groups on day 10 p.i. Th1 (CD4^+^IFN-γ^+^) cells (**a**), Th17 (CD4^+^IL-17^+^) cells (**b**), and Treg (CD4^+^CD25^+^Foxp3^+^) cells (**c**) were detected by flow cytometry. For Treg cell analysis, CD4^+^ cells were first gated. The results were expressed as mean ± SD (*n* = 4). *p < 0.05, **p < 0.01, two-tailed Student’s *t* test for unpaired data

Since lymphocytes are responsible for the initiation of EAN and tend to disappear in the peripheral nerves around day 13 p.i. [24], we chose day 10 p.i. as a time point to evaluate the lymphocyte response as reported previously [2]. On day 10 p.i., the rats from both groups were sacrificed and mononuclear cells were harvested and analyzed by flow cytometry (FC). Results showed a significant decrease in the percentages of Th1 cells (CD4^+^IFN-γ^+^, 0.78 ± 0.15% vs. 0.35 ± 0.06%, *p* < 0.01) and Th17 cells (CD4^+^IL-17^+^, 1.70 ± 0.55% vs. 0.93 ± 0.17%, *p* < 0.05) (Fig. 3a, b), accompanied by a significant increase in the percentage of Treg cells (CD4^+^CD25^+^Foxp3^+^) (5.63 ± 0.52% vs. 6.55 ± 0.49%, *p* < 0.05) (Fig. 3c), after 2-DG treatment.

**Fig. 4:**
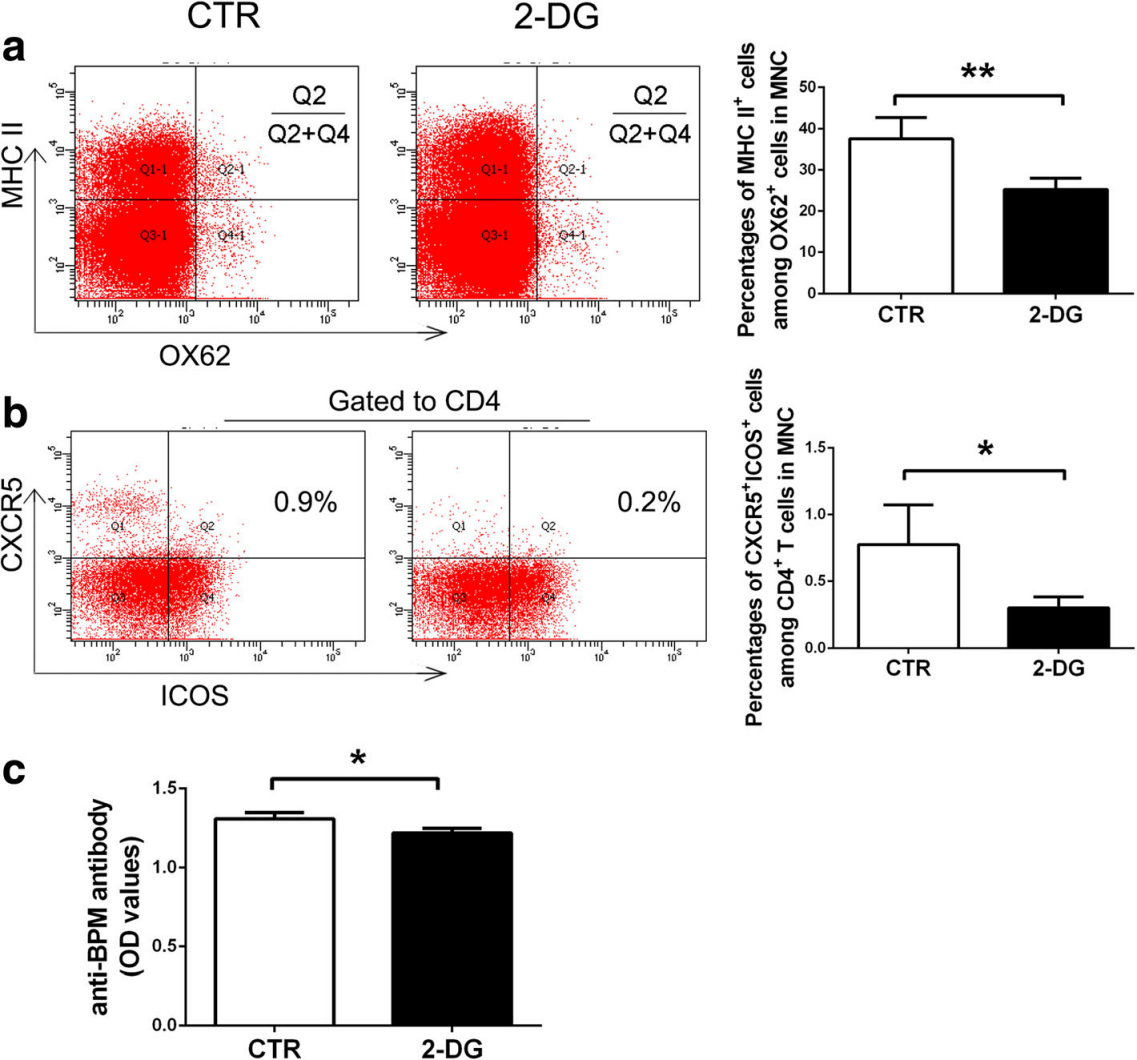
Preventive treatment of 2-DG suppressed humoral immune response on day 13 p.i. The expression of MHC II on OX62^+^ DC (**a**) and the percentages of Tfh (CD4^+^CXCR5^+^ICOS^+^) cells (**b**) were detected by flow cytometry. For Tfh cell analysis, CD4^+^ cells were first gated. The level of anti-BPM antibody (**c**) in the serum was determined by ELISA. The results are expressed as mean ± SD (*n* = 4). *p < 0.05, **p < 0.01, two-tailed Student’s *t* test for unpaired data

Apart from the cellular immune response, we further analyzed the humoral immune response on day 13 p.i. DC are vital for humoral immune response, and the maturation of DC could be indicated by the expressions of MHC II, CD80, and CD86. Thus, we analyzed these molecules with FC. 2-DG treatment significantly reduced the expression of MHC II (37.58 ± 5.20% vs. 25.28 ± 2.71%, *p* < 0.01) (Fig. 4a) on OX62^+^ DC, while no significant change was found in the expression of CD86 (data not shown). Follicular helper T cells (Tfh) are specialized effector T cells that assist B cell development to produce high-affinity immunoglobulins [25]. We found 2-DG significantly reduced the percentages of Tfh cells identified as CD4^+^CXCR5^+^ICOS^+^ cells (0.78 ± 0.30% vs. 0.30 ± 0.08%, *p* < 0.05) (Fig. 4b). Along with the reduction of Tfh cells, the level of anti-BPM antibody was also decreased (*p* < 0.05) (Fig. 4c).

### 2-DG halts the progression of ongoing EAN and improves histological changes

**Fig. 5:**
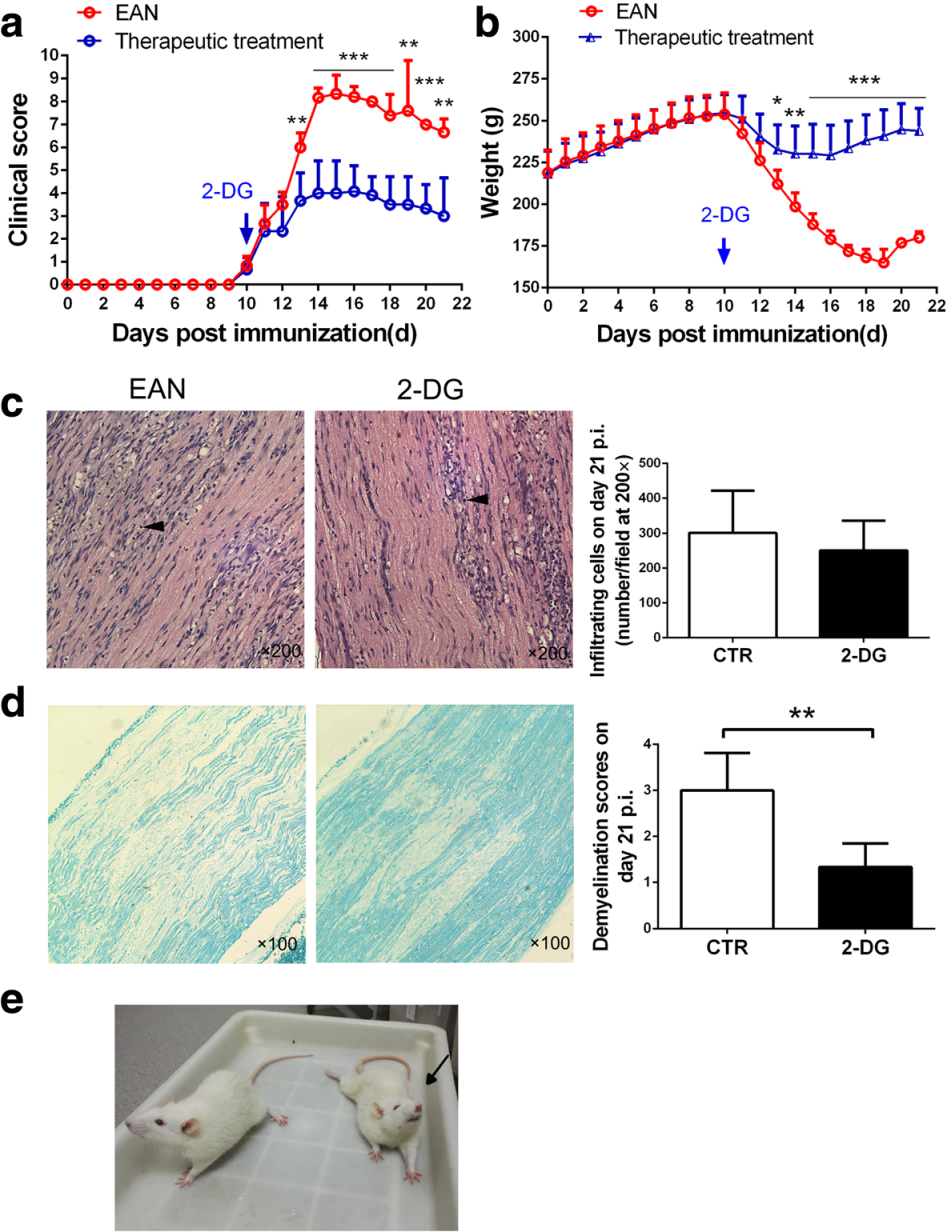
Therapeutic treatment of 2-DG reduced the severity of EAN. The rats were intraperitoneally injected with 2-DG (550 mg/kg) daily from day 10 p.i. (the neurological deficit appeared) to day 21 p.i. (all rats showed signs of recovery). *n* = 6 in each group (two rats in control group died of respiratory failure). Obvious improvement in the clinical scores (**a**) and the weight loss (**b**) were observed in 2-DG group. *p < 0.05, **p < 0.01, ***p < 0.001. **c** H&E staining of the sciatic nerves on day 21 p.i. (× 200 magnification, arrows denote the inflammatory mononuclear cells). (**d**) The demyelination was reduced, shown with LFB staining on day 21 p.i. (× 100 magnification). Mean histological scores were calculated as described in the methods and materials part (**p < 0.01) (*n* = 4 in control group; *n* = 6 in 2-DG group). **e** More serious weight loss and paralysis could be observed in the control rat (arrowed) than in the 2-DG-treated rat. The results are expressed as mean ± SD, two-tailed Student’s *t* test for unpaired data

For therapeutic treatment, 2-DG (550 mg/kg) was intraperitoneally injected once daily from the onset to the recovery stage of EAN (day 10–21 p.i.). The dose of 2-DG in our study was equivalently converted from the dose in human clinical trial [26]. As shown in Fig. 5a, 2-DG treatment nearly completely suppressed EAN progressing from day 13 to 21 p.i. The maximal clinical score was significantly reduced in 2-DG group (4.00 ± 1.41) than in the control group (8.33 ± 0.82) (*p* < 0.001). 2-DG treatment also significantly attenuated the loss of body weight from day 13 to 21 p.i. (Fig. 5b), with the minimum weight of 230.33 ± 16.74 g on day 14 p.i. while the minimum weight in the control group was 165.00 ± 8.15 g on day 19 p.i. A picture on day 15 p.i. showed a milder symptom in the 2-DG-treated rat than in the control rat (arrowed) (Fig. 5e). Therefore, the therapeutic treatment of 2-DG significantly reduced the severity of EAN.

The rats from both groups were killed on day 21 p.i. to remove sciatic nerves for histological analysis. H&E staining was used to evaluate inflammatory cell infiltration and LFB staining to show myelin. There was no significant difference of the number of infiltrated cells between two groups (Fig. 5c), while the demyelination was significantly reduced in 2-DG-treated group than in the control group (1.33 ± 0.52 vs. 3.00 ± 0.82) (*p* < 0.01) (Fig. 5d).

### Therapeutic treatment of 2-DG modulates immune response in vivo

**Fig. 6:**
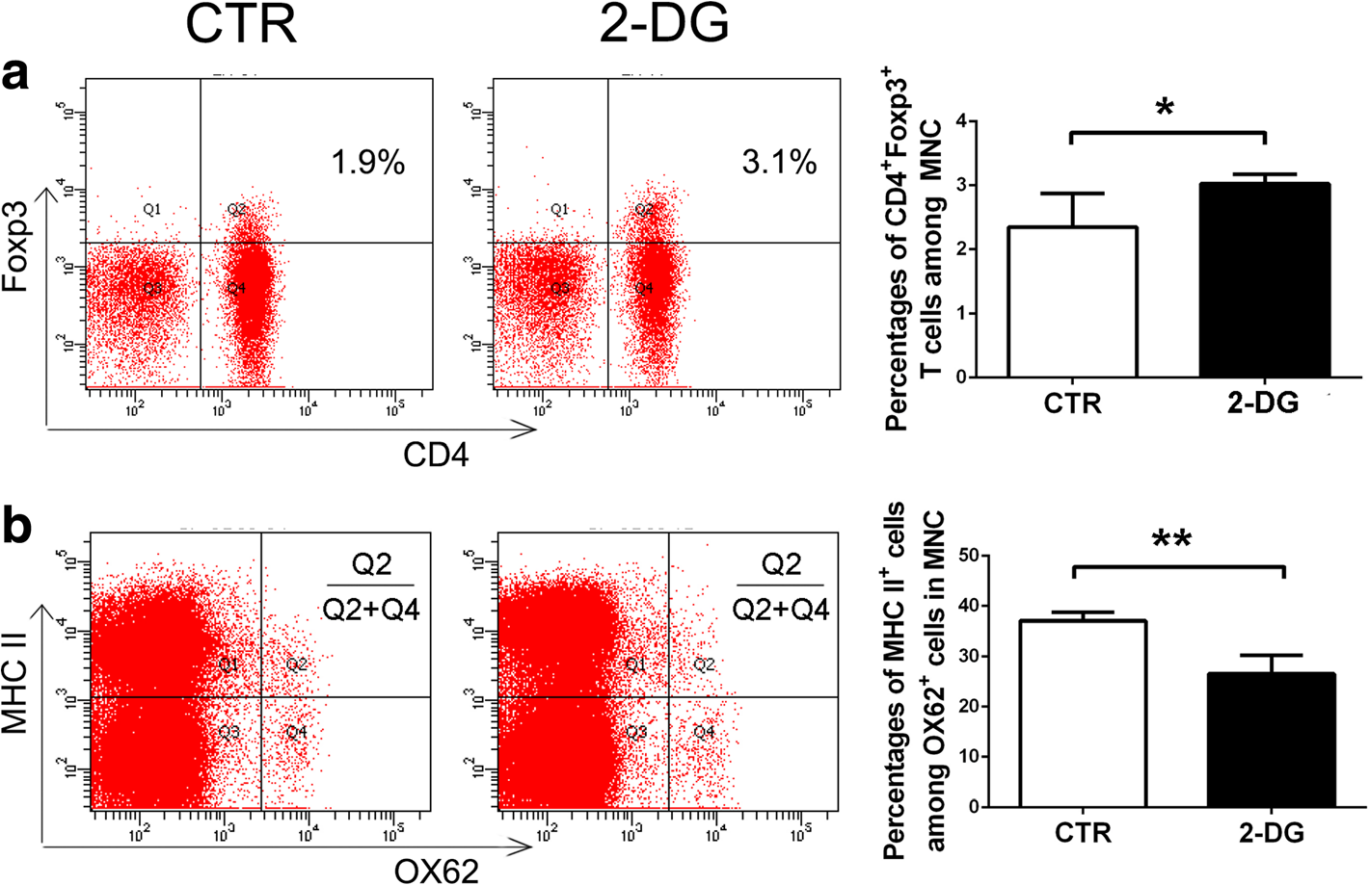
Therapeutic treatment of 2-DG increased Treg cells and decreased the maturation of DC in vivo. MNCs were harvested from both groups on day 14 p.i, when the symptoms peaked. The percentages of Treg (CD4^+^Foxp3^+^) cells (**a**) and the expression of MHC II (**b**) on OX62^+^ DC were determined by flow cytometry. The results were displayed in the corresponding graphs, expressed as mean ± SD (*n* = 4). *p < 0.05, **p < 0.01, two-tailed Student’s *t* test for unpaired data

On day 14 p.i., when the symptoms of EAN peaked, MNCs from the lymph nodes and the spleens were isolated separately and analyzed by FC again. Compared with the control group, there is no significant inhibition of Th1 or Th17 cells after therapeutic treatment with 2-DG (data not shown), while the percentages of Treg cells were increased (2.35 ± 0.53 vs. 3.03 ± 0.15%, *p* < 0.05) (Fig. 6a). The expression of MHC class II on OX62^+^ DC was also inhibited following 2-DG treatment (37.08 ± 1.68% vs. 26.55 ± 3.72%, *p* < 0.01) (Fig. 6b), but no significant change was observed in the expression of CD80 and CD86 on OX62^+^ DC in vivo (data not shown).

### 2-DG inhibits the LPS-stimulated maturation of dendritic cells in vitro

**Fig. 7:**
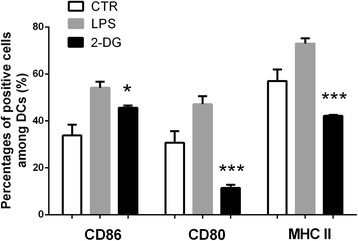
2-DG inhibited the maturation of dendritic cells in vitro. The expression of MHC II, CD80, and CD86 on the LPS-activated dendritic cells was inhibited by 2-DG (1 mM), determined by flow cytometry. The results are expressed as mean ± SD (*n* = 3). *p < 0.05, ***p < 0.001, one way ANOVA

To further explore the effects of 2-DG on DC maturation, we performed in vitro experiments. As shown in Fig. 7, 1 mM 2-DG significantly decreased the LPS-induced expression of MHC II, CD80, and CD86 on DC (*p* < 0.001, *p* < 0.001, *p* < 0.05 vs. LPS group, respectively).

### 2-DG inhibits phagocytosis and NO production of macrophage in vitro

**Fig. 8:**
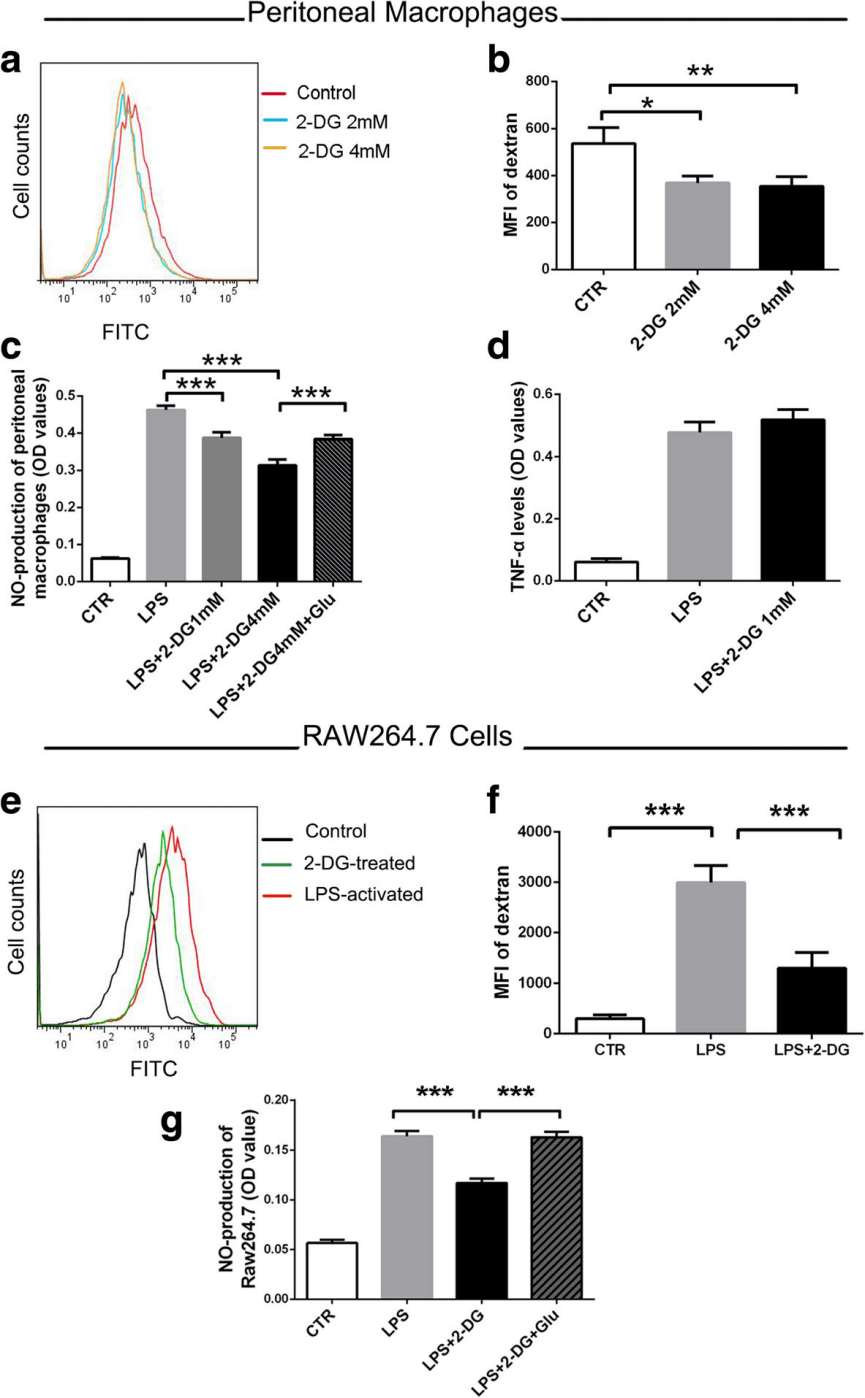
2-DG inhibited phagocytosis and NO production of macrophages in vitro. The phagocytosis of FITC-dextran was inhibited in the isolated peritoneal macrophages (**a**, **b**) and the LPS-activated RAW264.7 cells (4 mM 2-DG) (**e**, **f**), after 2-DG treatment, determined by flow cytometry. NO production in the LPS-stimulated peritoneal macrophages (**c**) and RAW264.7 cells (**g**) treated with different concentrations of 2-DG with or without glucose addition (glucose concentration increased 4 g/l, 8 g/l, respectively) were examined by Griess reagent. **d** TNF-α levels of the LPS-stimulated peritoneal macrophages were examined by ELISA. Results are expressed as mean ± SD (*n* = 3). *p < 0.05, **p < 0.01, ***p < 0.001, one way ANOVA

Phagocytic attack and pro-inflammatory mediator secretion by macrophages play a critical role in the pathogenesis of inflammatory neuropathies of EAN [27]. To assess the phagocytosis of macrophages, FITC-dextran (molecular weight 40,000) was used as a model antigen and its uptake was detected by FC. Data showed 2-DG significantly inhibited the uptake of dextran by the isolated peritoneal macrophages (Fig. 8a, b) and the LPS-activated RAW264.7 cells (Fig. 8e, f). Compared with the LPS group, NO production of peritoneal macrophages in 2-DG treatment group was significantly reduced in a dose-dependent manner and partly reversed by glucose addition (*p* < 0.001) (Fig. 8c). In RAW264.7 cells, the cell line of macrophages, the same trend of reduced NO production was observed after 2 mM 2-DG treatment (*p* < 0.001) (Fig. 8g) and reversed completely by glucose-high medium (Fig. 8g). Although TNF-α has a pivotal role in the pathogenesis of EAN and GBS [28], no significant change of its secretion by macrophage was observed after 2-DG (1 mM) treatment in vitro (Fig. 8d).

## Discussion

To reveal the role of glycolysis in the pathogenesis of GBS, we accessed the effects of glycolysis inhibition with 2-DG on EAN, a classic model of GBS. We found glycolysis inhibition with 2-DG not only inhibited the initiation but also prevented the progression of EAN. The underlying mechanisms might include suppressing Th1, Th17, and Tfh cell development, promoting Treg cell differentiation, inhibiting DC maturation, and decreasing the NO production and phagocytosis of macrophages. This study indicates that enhanced glycolysis contributes to the pathogenesis of EAN and provides a new target in GBS treatment.

EAN is characterized by the accumulation of activated T cells and macrophages in the peripheral nerve system. The rapid weight loss (Fig. 2), which cannot be completely explained by the disease-associated paralysis [29], suggests an abnormal metabolism in EAN. According to previous reports, after differentiation or activation, pro-inflammatory cells like Th1, Th17, DC, and macrophages undergo a metabolic shift away from OXPHOS towards aerobic glycolysis [4, 5]. Consistently, we also observed the enhanced glucose uptake, glucose consumption, and acid production in LPS-activated macrophages (Fig. 1). We believed the increased glycolysis concomitant with the differentiation of pro-inflammatory cells also existed in vivo and contributed to the pathogenesis of EAN. The effects of glycolysis inhibitor on EAN in our study supported this hypothesis.

We employed two treatment protocols in this study: preventive and therapeutic schemes. Firstly, the preventive treatment profoundly impeded the initiation of EAN, documented by the decreased clinical score, weight loss, and inflammatory cell infiltration especially CD68^+^ macrophages. In EAN, the decisive role of T lymphocytes in the initiation of EAN has been firmly established by adoptive transfer experiments [24]. Th cell subsets like Th1 and Th17 cells participate in the development of EAN by secreting pathogenic cytokines and recruiting and activating other inflammatory cells. These effector T cells utilize large amounts of glucose and high glycolysis to meet their energetic needs, while the principal anti-inflammatory cell, Treg cells, depends on lipid metabolism and could expand and function even without glucose [8]. The upregulation of glycolysis in T cell, controlled by the mTOR-HIF-1α pathway, is not just a consequence of differentiation, but rather a necessary step to facilitate differentiation [17]. In accordance with the report of mTOR inhibition in vitro [17], in our study, direct glycolysis inhibition with 2-DG in vivo also dampened the development of Th1 and Th17 cells while promoting the differentiation of Treg cells. The percentage changes of Th1 and Th17 cells were indeed not very much in the current study; however, the reduction rates were considerable. Considering the enormous amount of mononuclear cells in the lymph nodes, the absolute numbers of Th1 and Th17 cells might be reduced a lot, which resulted in the decreased Th1 and TH17 polarization.

Glycolysis not only participates in the cellular immunity but also contributes to the humoral immunity. The development of germinal center relies on adequate antigen presentation from antigen-presenting cells like DC and the assistance of Tfh cells. Both cells depend on glycolysis to mature or differentiate [16, 30]. In addition, glycolysis also supports the survival of cells in the oxygen-limited germinal center resulted from intense cell proliferation and poor vascularization [31]. In our study, glycolysis inhibition with 2-DG significantly suppressed the maturation of DC and the differentiation of Tfh cells. Meanwhile, the level of anti-BPM antibody was also reduced. The antibody deposition and complement activation are considered central for demyelination and conduction block in GBS [1, 32, 33]. These data implied that glycolysis contributed to the early events in the development of EAN, like T cell differentiation, expansion, and antibody secretion.

Then, we further investigated the effects of glycolysis inhibition with 2-DG on established EAN model. The severity of EAN was reduced by 2-DG even when administered after the onset of EAN, evidenced by the milder demyelination and less weight loss compared with the control group. As for the peripheral immune response, in line with the results of preventive treatment, the same trend of increased anti-inflammatory Treg cells was observed in 2-DG group. Meanwhile, the maturation of DC was also suppressed in vivo and in vitro. The percentages of Th1 and Th17 cells were not changed after therapeutic treatment with 2-DG, which might explain no significant difference of inflammatory cell infiltration in peripheral nerves between the two groups. These data indicated that other mechanism than the suppression of T cell differentiation may contribute to the protective effects of 2-DG.

Macrophages, the major cell population in the inflamed peripheral nerves of EAN, directly participated the local inflammation by phagocytic attack and secretion of pro-inflammatory mediators like NO, matrix metalloproteinases (MMPs), and TNF-α. According to previous report, the amount of cells bearing inducible nitric oxide synthase (iNOS) and TNF-α was parallel to the clinical symptoms of EAN [34]. In response to inflammatory stimuli, iNOS produces amounts of nitric oxide (NO), which is considered as a key process in the demyelination in both peripheral and central nervous system [35–38]. NO exerts pro-inflammatory functions via cytotoxicity [39], mitochondrial respiration inhibition [40], and the mediation of cytokine-dependent tissue damage process [41]. Inhibition of iNOS has been proven to ameliorate EAN [35]. In our study, glycolysis inhibition with 2-DG decreased the NO production of both LPS-stimulated peritoneal macrophages and RAW264.7 cells in vitro. The decrease partly resulted from the competitive inhibition of glucose metabolism by 2-DG, since higher glucose could reverse it. Our data were in agreement with a previous research, which showed that glucose metabolism was involved in the regulation of iNOS expression [42]. However, the secretion of the major pathogenic cytokine in EAN, TNF-α [28], was not influenced by 2-DG in vitro. This result indicated that TNF-α might not be the target of 2-DG, as the previous studies revealed [10, 23]. Additionally, phagocytic attack was another vital mechanism underlying the myelin destruction by macrophages [43, 44], especially at the amplification stage of EAN. We found 2-DG treatment significantly decreased the phagocytosis of activated macrophages in vitro. Apart from NO production and phagocytosis, glycolysis inhibition could also suppress the migration of macrophages [45] which could contribute to the curative effects of 2-DG in EAN, although we did not assess in our study.

After glycolysis inhibition in vivo, cells of the body mainly depend on ketone body to meet the energy needs. Ketogenic diet is high-fat diet with adequate protein and low carbohydrates, established as a treatment to control refractory epilepsy by reducing glycolysis in the brain cells [46]. Animal experiment of multiple sclerosis also showed inhibition of the neuro-inflammation by ketogenic diet [47]. Whether simple ketogenic diet, instead of glycolysis inhibitor, could obtain good curative effects on EAN or GBS will need to be explored in the future.

## Conclusion

In this study, we showed that glycolysis inhibition with 2-DG significantly inhibited both the initiation and the progression of EAN. The underlying mechanism included dampening Th1, Th17, and Tfh development, promoting Treg differentiation, inhibiting DC maturation, and decreasing the NO production and phagocytosis of macrophages. Our data indicated that enhance glycolysis participated in the pathogenesis of EAN and suggested that a glycolysis inhibition strategy might be a novel therapeutic intervention for GBS.

## Abbreviations

2-DG: 2-Deoxy-d-glucose
2-NBDG: 2-(*N*-(7-Nitrobenz-2-oxa-1,3-diazol-4-yl) amino)-2-deoxyglucose
AMPK: AMP-activated protein kinase
BPM: Bovine peripheral myelin
CTR: Control
DC: Dendritic cells
EAN: Experimental autoimmune neuritis
FC: Flow cytometry
GBS: Guillain-Barré syndrome
GM-CSF: Granulocyte macrophage colony-stimulating factor
H&E: Hematoxylin-eosin
HIF-1α: Hypoxia-inducible factor-1α
HK: Hexokinase
IFN-γ: Interferon gamma
iNOS: Inducible nitric oxide synthase
LFB: Luxol fast blue
LPS: Lipopolysaccharides
MFI: Mean fluorescence intensity
mTOR: Mammalian target of rapamycin
NO: Nitric oxide
OD: Optical density
OXPHOS: Oxidative phosphorylation
SLE: Systemic lupus erythematosus
Tfh: Follicular helper T cells
Th: T helper
Tregs: Regulatory T cells
